# Editorial: Induced resistance and priming against pests and pathogens

**DOI:** 10.3389/fpls.2022.1075783

**Published:** 2022-11-16

**Authors:** Michele Perazzolli, Jurriaan Ton, Estrella Luna, Brigitte Mauch-Mani, Maria L. Pappas, Michael R. Roberts, A. Corina Vlot, Víctor Flors

**Affiliations:** ^1^ Center Agriculture Food Environment (C3A), University of Trento, San Michele all’Adige, Italy; ^2^ Research and Innovation Centre, Fondazione Edmund Mach, San Michele all’Adige, Italy; ^3^ School of Biosciences, University of Sheffield, Sheffield, United Kingdom; ^4^ School of Biosciences, University of Birmingham, Birmingham, United Kingdom; ^5^ Laboratoire de Biologie Moléculaire et Cellulaire, Institute of Biology, Université de Neuchâtel, Neuchâtel, Switzerland; ^6^ Department of Agricultural Development, Faculty of Agricultural and Forestry Sciences, Democritus University of Thrace, Orestiada, Greece; ^7^ Lancaster Environment Centre, Lancaster University, Lancaster, United Kingdom; ^8^ University of Bayreuth, Faculty of Life Sciences: Food, Nutrition and Health, Chair of Crop Plant Genetics, Kulmbach, Germany; ^9^ Biochemistry and Molecular Biology Section, Department of Biology, Biochemistry and Natural Sciences, Universitat Jaume I, Castellón, Spain

**Keywords:** induced resistance, plant defense, plant immunity, plant-microbe interaction, priming

## Introduction

Due to the rapidly changing climate and increasingly restrictive regulations on the use of pesticides, there is an urgent need to discover and develop new and more sustainable strategies of crop protection that meet the present and future needs of a growing world population. Fundamental research on plant-microbe and plant-insect interactions – both pathogenic and beneficial – is of key importance to gain a better molecular, physiological and ecological understanding of these complex interactions and so generate the tools necessary to develop new crop protection strategies.

Induced resistance (IR) develops after treatment of plants with pathogens, pests, beneficial microorganisms, chemical agents, physical wounding, or herbivory. Plants exposed to such stimuli increase their level of basal resistance against future attacks compared to non-stimulated plants. IR is often based on a priming of basal defense mechanisms, which enables a faster and/or stronger defense response upon secondary challenge. Given its long-lasting nature and broad-spectrum effectiveness, IR has long been recognized for its value in integrated pest and disease management approaches. This Research Topic highlights the latest advances in research on IR and priming presented at the IOBC-PR-IR2022 conference in Sheffield, UK, from 4^th^ to 7^th^ April 2022, which is organized by the working group of the International Organization for Biological Control. In addition to reviewing the scientific significance of this work, we discuss future challenges in IR research and the potential application of IR in future crop protection strategies.

## Conference of the IOBC-PR-IR working group

In addition to the contributions to the current guest issue, this editorial aims to briefly highlight the main issues presented and discussed by delegates attending IOBC-WPRS PR-IR 2022: ‘Priming the Future for Healthy Plants’ held from 4-th to 7^th^ April 2022 in Sheffield, UK, and partially sponsored by Frontiers in Plant Science. This conference was a joint meeting of the PR proteins special interest group and the ‘Induced Resistance in Plants Against Insects and Diseases’ working group of the International Organisation for Biological Control (IOBC). It is the latest in a series of IOBC meetings originally initiated in 2001 to provide a forum for researchers on plant responses to microbial pathogens and invertebrate herbivores to exchange information and discuss the latest ideas on IR. The focus is on fundamental science, but with a view to the potential to exploit new understanding for crop protection. The meeting covered topics ranging from mechanisms for the initial perception of pests and pathogens and signalling pathways for IR to how multitrophic interactions with microbiomes and natural enemies influence the relationships between plants and their pests and pathogens. Topics were grouped in a series of themes starting with ‘Perception and Signalling’ with updates from Jurgen Zeier (Germany) and Christine Faulkner (UK) on different mechanisms for systemic signalling during IR, and insights into the new field of plant recognition of damaged-self molecules such as extracellular DNA from Martin Heil (Mexico) and Leila Rassizadeh (Spain). Sessions on early responses to priming and IR stimuli were followed by ‘Cell-wall mediated immunity’ and ‘Transcriptional control’ of priming highlighting the main events downstream perception. On transcriptional control, there were several talks covering large-scale network construction and identification of genes by association mapping based on collections of ‘omics data (Fumi Katagiri, USA; Saskia van Wees, Netherlands; Andrea Harper, UK; Stephen Spöel, UK). Understanding of long-lasting intra- and transgenerational priming was covered in deep by a series of talks focusing on ‘Epigenetic regulation’. This session provided exciting insights into the emerging knowledge of how long-term memories of stress may be encoded *via* DNA methylation and histone modifications (e.g. Melissa Mageroy, Norway and Anikó Meijer, Belgium).

Comprehension of the role of the soil microbiome in plant immunity was revealed as a tool with enormous potential to be exploited in sustainable agroecosystems. Noteworthy, plant-microbiome interactions have additional complexities. Firstly, the stimulus is another biological system adding variability, and secondly, the ‘Tri-trophic and community interactions’ are closer to real field conditions. In these multi-way interactions studies, plant immune responses are not always the same as found in two-way plant-pathogen or plant-insect studies. Outstanding studies presented by Ainhoa Martinez-Medina (Spain), Christelle Robert, (Switzerland) and Sharon Zytynska (UK), among other presentations, showed how plant interactions with herbivores and pathogens can be affected by microbial communities or specific beneficial fungi including arbuscular mycorrhiza, by herbivore natural enemies, and even by neighbouring plants. Despite the fundamental research aspects covered during the conference, an insight into how microbes can be developed into commercial biocontrol solutions was also presented (Sjoerd van der Ent, Koppert Biological Systems, Netherlands). Concluding remarks about ‘Past, Present and Future of Induced Resistance Research’ were presented by Uwe Conrath (Germany) and Maria Jose Pozo (Spain). They outlined their views on the major landmarks in IR research and the potential for translating our knowledge into agricultural crop protection solutions.

## Recent updates on mechanisms of induced resistance and priming

### Phytohormonal pathways

The phytohormones salicylic acid (SA), ethylene (ET), jasmonic acid (JA) and n-hydroxy-pipecolic acid (NHP) play crucial roles in mediating IR and plant defense against pests and pathogens. However, their effectiveness is often pathosystem-dependent. For instance, the role of methyl-SA in IR has been reported to vary between plant species (Vlot et al., 2008). In sweet orange (*Citrus sinensis*), overexpression of a SA-dependent methyltransferase (SAMT) resulted in increased production of methyl-SA and reduced severity to citrus canker caused by *Xanthomonas citri* subsp. *Citri* (Nascimento et al., 2022). These SAMT-overexpressing plants also showed transcriptional modulation of SA-related genes prior inoculation, indicating that signaling events activated by high levels of MeSA can prompt the plant to respond more efficiently to pathogen attack and to activate immune responses at both primary and secondary infection sites (Nascimento et al., 2022).

### Boosting the cost-benefit balance of IR agents

A major obstacle against commercial exploitation of chemical IR agents is that they can have negative effects on plant growth and seed set when applied in higher concentrations or render plants more susceptible to other stresses through negative signaling cross-talk. Thus, to safely apply these agents in commercial crop protection initiatives, it is important to uncouple IR efficiency from their detrimental side-effects (Yassin et al., 2021). Following foliar application of Cucurbitaceae COld Peeling Extract (CCOPE) derived from peels of melon (*Cucumis melo* var. *cantalupensis*; mCOPE; De Kesel et al., 2022), roots of rice (*Oryza sativa*), activate phytohormone signaling mediated by ET and JA, as well as reactive oxygen species (ROS). This mCOPE agent combines an IR-triggering capacity with direct nematicidal effects against the root-knot nematode *Meloidogyne graminicola* (De Kesel et al., 2022). Importantly, the agent had no negative side effects on plant growth, nor does it compromise resistance to necrotrophic pests or pathogens, suggesting promising application potential of this agro-industrial waste stream extract as a plant resistance inducer (De Kesel et al., 2022). Another strategy to uncouple IR from plant stress is to study the underpinning mechanisms causing the stress responses. For instance, over-expressing the BABA receptor gene *IBI1* not only increases BABA-IR efficacy but also mitigates against BABA-induced stress (Luna et al., 2014). The LHT1 transporter of the beta-amino acid priming agents BABA and R-beta-homoserine (RBH), determines the balance between IR efficiency and growth repression by BABA and RBH (Tao et al., 2022). By using *IBI1* and *LHT1* as breeding targets it is possible to select for new crop varieties that develop optimal levels of beta-amino acid-IR without side-effects on growth and reproduction.

### Epigenetic control of IR

Priming activated by resistance inducers is not only functional in the next hours following stimuli application but can be also long-lasting and involve epigenetic regulation of stress-responsive genes. Catoni et al. (2022) found in tomato a reduction in cytosine methylation in gene promoters and DNA transposons of tomato plants primed for pathogen resistance by treatment with β-aminobutyric acid (BABA). Similarly, Wilkinson et al. (2022) reported in Arabidopsis that long-lasting JA-IR against herbivore feeding was associated with reduced cytosine methylation at *AtREP2* transposons. However, in both cases, the majority of priming-related changes in DNA methylation did not occur in the proximity of primed genes, suggesting that methylation may affect priming *via trans*-acting mechanisms. The implication of epigenetic mechanisms in the regulation of long-term IR and defence gene priming offers opportunities to directly manipulate the epigenetic make-up of plants and select for constitutively primed plants. However, more adjustable and precise epi-mutagenesis strategies are needed, allowing for the introduction of sufficient epigenetic change to induce heritable variation in primed resistance, whilst preventing overstimulation causing sterile/lethal phenotypes (Cooper and Ton, 2022).

### IR by plant-beneficial microbes

An efficient strategy to combine IR with growth promotion is the use of beneficial soil microbes as IR agents. Mycorrhizal fungi are plant-beneficial fungi that can induce plant resistance against multiple biotic stressors and can supply plant growth with inorganic nutrients and water in exchange for carbohydrates. In this context, Hampejsová et al. (2022) showed that mycorrhizal inoculation (*Tulasnella calospora*) can increase the content of antifungal compounds, promote flavonoid biosynthesis, and increase the formation of lipid droplets and the associated production of oxylipins in orchid tubers (*Dactylorhiza* sp.). Moreover, tubers of inoculated plants accumulated higher amounts of antifungal compounds (e.g., phenolics, alkaloid calystegine B2, and dihydrophenanthrenes) with inhibitory effects against a pathogenic oomycete (*Phytophthora cactorum*; Hampejsová et al., 2022). Likewise, citrus plants (*Citrus aurantium*) colonized by the mycorrhizal fungus (*Rhizophagus irregularis*) displayed reduced levels of leaf damage and oviposition rates of the two-spotted spider mite (*Tetranychus urticae*) compared to control plants (Manresa-Grao et al., 2022). Mycorrhizal citrus plants showed upregulated expression of oxylipin-related genes (*LOX-2* and *PR-3*) and increased content of stress-related oxylipins such as JA, 12-oxo phytodienoic acid, and JA-isoleucine. Interestingly, specific groups of primary and secondary metabolites like amino acids, oxocarboxylic acids, and phenylpropanoids displayed primed accumulation following spider mite infestation (Manresa-Grao et al., 2022), amounting to the growing evidence that mycorrhiza-IR is based on the priming of multiple defense pathways.

### Egg-IR against herbivory

IR against herbivores can be triggered by oviposition, which provides plants with ample opportunity to develop a suitable IR response at the time the herbivore’s eggs hatch (Reymond, 2013). Recent work identified that phosphatidylcholines in eggs from the specialist herbivore *Pieris brassicae* activate IR-related immune responses (Stahl et al., 2020). Comparative transcriptomic analysis of Arabidopsis plants showed that spider mite (*T. urticae*) and lepidopteran (*Pieris brassicae*) oviposition elicit specific responses showing a time-dependent succession of unique processes (Ojeda-Martinez et al., 2022). In particular, transcriptional regulation involved defence-related processes (e.g., SA, JA, ROS, glucosinolates), cell wall rearrangements, abiotic stress responses, and energy metabolism, suggesting that specific pathways are activated to optimize the IR response in accordance to the herbivore attacker (Ojeda-Martinez et al., 2022).

Since IR increases the efficiency of basal resistance mechanisms, studying genetic variation in the effectiveness of basal defence responses would allow for the selection of varieties with increased IR capacity. A combined transcriptome and metabolome analysis of resistant and susceptible quinoa cultivars (*Chenopodium quinoa* Willd.) highlighted key metabolites (e.g. alkaloids and phenolic acids) and genes (bZIP transcription factors) responsible for the differential regulatory mechanisms against insects (*Spodoptera exigua*; Liu et al., 2022). Future research will have to point out whether these biochemical markers of basal defence responses can be used as markers for IR capability.

## Outlook and future challenges

Fundamental research continues to provide new insights in plant immunity in plants, generating new opportunities to translate this fundamental knowledge into application. This translation will require applied studies that take this basic knowledge and apply it in commercial crop production systems. This guest issue about the IOBC-PR-IR2022 conference on IR illustrates the pathways through which basic knowledge from the IR research field can be integrated in crop protection strategies, as part of a wider Integrated Pest Management portfolio. This innovation pipeline must explore the use of new priming stimuli as well as long-term effects on plants and their offspring, which will inform the sustainable agriculture of the future.

## Author contributions

All authors listed have made a substantial, direct, and intellectual contribution to the work and approved it for publication.

## Acknowledgments

The organising committee of JT (University of Sheffield, UK), VF (University Jaume I, Spain), MR (Lancaster University, UK) and EL (University of Birmingham, UK), would like to give thanks to all our sponsors: International Organization for the Biological Control, British Society for Plant Pathology, Crop Health and Protection UK Agri-Tech Innovation Centre (CHAP), Society of Experimental Biology (SEB), and the Company of Biologists.

## Conflict of interest

The authors declare that the research was conducted in the absence of any commercial or financial relationships that could be construed as a potential conflict of interest.

## Publisher’s note

All claims expressed in this article are solely those of the authors and do not necessarily represent those of their affiliated organizations, or those of the publisher, the editors and the reviewers. Any product that may be evaluated in this article, or claim that may be made by its manufacturer, is not guaranteed or endorsed by the publisher.
